# NDRG2 as a marker protein for brain astrocytes

**DOI:** 10.1007/s00441-014-1837-5

**Published:** 2014-05-10

**Authors:** Gabriele Flügge, Carolina Araya-Callis, Enrique Garea-Rodriguez, Christine Stadelmann-Nessler, Eberhard Fuchs

**Affiliations:** 1 Clinical Neurobiology Laboratory, German Primate Center, Leibniz Institute for Primate Research, Kellnerweg 4, Göttingen, 37077 Germany; 2 DFG Research Center for Molecular Physiology of the Brain, University of Göttingen, Göttingen, Germany; 3 Clinical Neurobiology Laboratory, German Primate Center, Leibniz Institute for Primate Research, Göttingen, Germany; 4 Department of Neuropathology, Medical School, University of Göttingen, Göttingen, Germany; 5 Department of Neurology, Medical School, University of Göttingen, Göttingen, Germany

**Keywords:** Astrocyte, NDRG2, GFAP, S100ß, Reactive astrocyte

## Abstract

The protein NDRG2 (N-myc downregulated gene 2) is expressed in astrocytes. We show here that NDRG2 is located in the cytosol of protoplasmic and fibrous astrocytes throughout the mammalian brain, including Bergmann glia as observed in mouse, rat, tree shrew, marmoset and human. NDRG2 immunoreactivity is detectable in the astrocytic cell bodies and excrescencies including fine distal processes. Glutamatergic and GABAergic nerve terminals are associated with NDRG2 immunopositive astrocytic processes. Müller glia in the retina displays no NDRG2 immunoreactivity. NDRG2 positive astrocytes are more abundant and more evenly distributed in the brain than GFAP (glial fibrillary acidic protein) immunoreactive cells. Some regions with very little GFAP such as the caudate nucleus show pronounced NDRG2 immunoreactivity. In white matter areas, NDRG2 is less strong than GFAP labeling. Most NDRG2 positive somata are immunoreactive for S100ß but not all S100ß cells express NDRG2. NDRG2 positive astrocytes do not express nestin and NG2 (chondroitin sulfate proteoglycan 4). The localization of NDRG2 overlaps only partially with that of aquaporin 4, the membrane-bound water channel that is concentrated in the astrocytic endfeet. Reactive astrocytes at a cortical lesion display very little NDRG2, which indicates that expression of the protein is reduced in reactive astrocytes. In conclusion, our data show that NDRG2 is a specific marker for a large population of mature, non-reactive brain astrocytes. Visualization of NDRG2 immunoreactive structures may serve as a reliable tool for quantitative studies on numbers of astrocytes in distinct brain regions and for high-resolution microscopy studies on distal astrocytic processes.

**Electronic supplementary material:**

The online version of this article (doi:10.1007/s00441-014-1837-5) contains supplementary material, which is available to authorized users.

## Introduction

There is an increasing interest in the role of glia in brain pathology, e.g., with respect to psychiatric disorders (Banasr et al. 2010; Czéh et al. 2013). In the hippocampus of patients with major depression (MD), glia cells and neurons are more densely packed than in healthy controls (Stockmeier et al. 2004). To analyze brains of patients post-mortem, antibodies against astrocytic marker proteins such as GFAP (glial fibrillary acidic protein) have been used in previous studies. GFAP immunoreactivity was found to be significantly reduced in hippocampal regions CA1 and CA2 as well as in the left orbitofrontal cortex of MD patients (Muller et al. 2001; Miguel-Hidalgo et al. 2010). However, since GFAP is a structural protein of intermediate filaments and as such constitutes only approximately 15 % of the total astrocytic volume, concentrations of this protein might not always be directly related to the numbers of astrocytes present in the respective brain region (Bushong et al. 2002; Lavialle et al. 2011). Moreover, GFAP expression is enhanced under pathological conditions that are accompanied by gliosis. This may lead to the detection of high GFAP concentrations in psychiatric patients with a gliosis, which may not necessarily be related to the respective psychiatric disease. Therefore, additional glial marker proteins are needed as tools for the visualization of glial cells.

It has been shown that antibodies against the cytosolic protein NDRG2 label brain glia cells (Okuda et al. 2008). *Ndrg2* belongs to the family of N-myc downregulated genes and is expressed in a variety of tissues throughout the body and central nervous system including glia (Okuda and Kondoh 1999; Wielpütz et al. 2007; Zhang et al. 2006). Functions of *Ndrg2* still remain to be elucidated but the gene has attracted particular attention because numerous studies have indicated that the NDRG2 protein is a tumor suppressor (Hwang et al. 2011). Transfection of human glioblastoma with *Ndrg2* cDNA reduced cell proliferation while low NDRG2 has been detected in colon carcinoma (Kim et al. 2009; Deng et al. 2003). However, *Ndrg2* mRNA was also found in many brain regions of healthy rats (Nichols 2003). Analysis of the fetal mouse revealed NDRG2 protein after embryonic day 13.5 with an increase in expression during the late stages of development (Hu et al. 2006). This developmental profile coincides with data indicating that NDRG2 plays a role in cell differentiation and studies in fetal mice have shown that a lack of *Ndrg2* leads to vertebral homeosis (Okuda et al. 2008; Zhu et al. 2012). Since adenovirus-mediated NDRG2 over-expression reduced the number of 5-bromo-2′-deoxy-uridine-incorporating cells in cultured astrocytes and enhanced the amount of F-actin, it was proposed that the gene suppresses proliferation and stabilizes morphology of cells (Takeichi et al. 2011). Our previous article showed that NDRG2 protein expression is enhanced in the hippocampus of chronically stressed rats (Araya-Callís et al. 2012). The aim of the present study is to analyze in which type of astrocytes the protein is expressed by comparing the pattern of NDRG2 immunoreactivity with that of other astrocytic marker proteins in mouse, rat, tree shrew, marmoset and human.

## Materials and methods

### Experimental animals

Five adult male C57BL/6 mice (22 ± 2 g) were obtained from The Jackson Laboratory (http://www.jax.org/). Five adult male Wistar rats (225 ± 6 g) were obtained from Harlan Winkelmann, Borchen, Germany. Five adult tree shrews (*Tupaia belangeri*), 3 females, 2 males, 190 ± 5 g, were obtained from the breeding colony at the German Primate Center, Göttingen, Germany. Housing of tree shrews has been described before (Fuchs and Corbach-Söhle 2010). Three adult marmoset monkeys (*Callithrix jacchus*), 1 female, 2 males (350–480 g) were obtained from the breeding colony at the German Primate Center. Housing of marmosets has been described before (Mitura et al. 2012). Two marmosets received a brain lesion with 6-hydroxydopamine (6-OHDA) (see below). All animal experiments were performed in accordance with the European Commission directive 2010/63/EU (from September 22, 2010) and were approved by the Lower Saxony Federal State Office for Consumer Protection and Food Safety, Germany.

### Cortical lesion

To analyze NDRG2 at a glial scar induced by physical trauma, we used brain sections from marmosets that had received 6-OHDA lesions in the caudate nucleus within a project not related to the present study. The neocortices of these animals displayed glial scars that were primarily caused by the penetrations of the injection needles. Two male marmosets received unilateral 6-OHDA lesions using a modified protocol based on Annett et al. (1992, 1995). The surgery was performed under deep anesthesia. As initial medication, animals received alphaxalon, diazepam and glycopyrronium bromide as described before (Ribic et al. 2011). After intratracheal intubation, they were inhaled with N_2_/O_2_ (70/30) and isoflurane (0.5–1.5 %) using a respirator (Animal Respirator; TSE, Bad Homburg, Germany). 6-hydroxydopamine hydrobromide (H-8523; Sigma-Aldrich, Steinheim, Germany) was dissolved in 0.01 % ascorbate saline and the toxin (5 μg in 3.0 μl) was injected stereotaxically into the caudate nucleus, at 6 sites in one hemisphere. Coordinates were determined with a marmoset brain atlas (Stephan et al. 1980); first site, AP +8.0, L +2.5, V +12.5; second site, AP +8.0, L  +3.5, V +13.5; third site, AP +9.0, L +2.5, V +12.0; fourth site, AP +9.0; L +3.5, V +13.0; fifth site, AP +10.0, L +2.25, V +12.0 and sixth site, AP +10.0, ML +3.25, V +13.0). The injections were performed with glass capillaries at a rate of 0.5 μl/min using Nano injector 2000 (WPI, Berlin, Germany). After each injection, the capillary remained in place for 4 min. After the surgery, the monkeys were kept in a warm incubator until they were well enough to be returned to their home cages. They were treated with meloxicam (0.2 mg/kg i.m.; Metacam^®^; Boehringer Ingelheim, Germany) for 3 days and with enrofloxacin (5 mg/kg i.m.; Baytril^®^; Bayer, Germany) for 5 days. 19 weeks after the surgery, the animals were sacrificed and their brains perfused as described below.

### Tissue preparation and perfusion

For fixation of the brains, rodents and tree shrews were deeply anesthetized with a mixture of GM II (Göttinger Mischung II: xylazine, 50 mg/mL; ketamine, 10 mg/mL; atropine, 0.1 mg/mL). Two male marmosets were anesthetized with GM II and received, after loss of consciousness, an intraperitoneal injection of ketamine (400 mg/kg body weight). Bodies were transcardially perfused with cold (4 ºC) saline (0.9 % NaCl) for 2 min (mice), 3 min (rats, tree shrews) or 5 min (marmosets). Subsequently, for fixation of the brains, cold (4 ºC) 4 % paraformaldehyde (PFA) in 0.1 M phosphate buffer, pH 7.2, was infused for 5 min (mice), 10 min (rats, tree shrews) or 15 min (marmosets). The heads were post-fixed in fresh 4 % PFA at 4 ºC overnight. The following day, brains were removed from the skulls and immersed in 30 % sucrose in PBS (phosphate buffered saline: 0.137 M NaCl, 2.7 mM KCl, 4.3 mM Na_2_HPO_4_ x 12H_2_O, 1.4 mM KH_2_PO_4_; pH 7.2) and incubated at 4 ºC for 2 days (for cryoprotection). Pieces of the brains were then frozen on dry ice and stored at −80 ºC until coronal sectioning was performed on a Leica cryostat (CM3050S; Leica Biosystems, Nussloch, Germany) at a thickness of 40 μm. Anatomical levels were chosen with brain atlases for the different species (mouse, Paxinos and Franklin 2008; rat, Paxinos and Watson 1986; tree shrew, Tigges and Shanta 1969; marmoset, Stephan et al. 1980).

Retinae were dissected from a female marmoset that had not been perfused. Euthanasia was performed with an overdose of GMII and ketamine as indicated above. Eyes were dissected immediately post-mortem and eye balls were frozen over liquid nitrogen. Cryostat sections (40 μm) were generated from the frozen eye balls and mounted on glass slides (Histobond; Marienfeld, Lauda-Königshofen, Germany) directly before the immunocytochemical treatment. Sections were mounted on glass slides and fixed with phosphate-buffered 4 % PFA for 2 min. The immunofluorescence experiments were performed as described below.

Human brain stem sections were derived from autopsy material generated and analyzed before in the Department of Neuropathology, University Medical Center, Göttingen (Singh et al. 2013). Specimen were post-fixed in phosphate buffered 4 % PFA, and were immersed in 30 % sucrose in PBS for cryoprotection and subsequent cutting in a cryostat (40-μm sections).

### Immunohistochemistry for light microscopy

Cryostat sections from perfusion fixed brains were immunohistochemically processed. Sections were washed in PBS and incubated in 1 % H_2_O_2_ (in PBS) for 30 min to inactivate endogenous peroxidase activity. All following washing steps were performed 3 times, 10 min each, in PBS. To block potential nonspecific antibody binding, sections were then incubated in 3 % NGS (normal goat serum; Vector Laboratories, Burlingame, CA, USA) in PBS with 0.3 % Triton X-100, for 1 h. Subsequently, sections were incubated with either the mouse polyclonal anti-NDRG2 antibody (H00057447-A01; Abnova, Taiwan, http://www.abnova.com; working dilution 1:1,000) or goat polyclonal anti-NDRG2 (sc-19468; Santa Cruz Biotechnology Inc., http://www.scbt.com, working dilution 1:1.000) in PBS-NGS-T (1 % normal goat serum, 0.3 % Triton X-100 in PBS) overnight at 4 ºC. After washing, the sections were incubated with secondary antibodies: biotinylated goat anti mouse IgG (BA-9200, Biozol; Diagnostica Vertrieb, Eching, Germany, working dilution 1: 400) or biotinylated horse anti goat IgG (BA-9500, Biozol, working dilution 1:400) in PBS-NGS-T, for 2 h. Subsequently, the sections were washed and incubated with the avidin-biotin complex (ABC Kit; Vector Laboratories) in PBS with 3 % NGS, for 1.5 h, according to the producer’s instructions. Finally, antibody binding was visualized with the chromogen 3,3′-diaminobenzidine (DAB Peroxidase Substrate Kit; Vector Laboratories) 0.025 %, with 0.01 % H_2_O_2_ as a catalytic agent. The sections were then washed, mounted on glass slides and left to dry overnight at 37 ºC. Dry sections were cleared in xylene and cover slipped with mounting medium (Eukitt; Sigma-Aldrich). Sections were examined with an Axiophot II microscope using a Plan-Apochromat ×63 oil immersion objective (NA 1.4) (Carl Zeiss Microimaging, Göttingen, Germany).

### Immunofluorescence and confocal microscopy

Cryostat sections (40 μm) from perfusion fixed brains were processed in the immunofluorescence experiments. Primary antibodies against the following antigens were used: *NDRG2*, mouse (ms) polyclonal (pc) antibody (ab) (# H00057447-A01; Abnova), working dilution 1:1,000; *NDRG2*, goat pc ab (# sc-19468; Santa Cruz Biotechnology), 1:1,000; *aquaporin 4* (AQP4) rabbit (rb) pc ab (# AB3594; Millipore/Chemicon, http://www.millipore.com), 1:1,000; *GFAP*, ms mc ab (# G 3893; Sigma-Aldrich), 1:5,000; *Iba1*, rb pc ab (019–19741; Wako), 1:2,000; *MAP2 (microtubule-associated protein 2)*, rb pc ab (AB5622; Chemicon), 1:1,000; *nestin*, ms mc ab (# 312011; Synaptic Systems, Göttingen, Germany), 1:1,000; *NG2*, rb pc ab (# AB5320; Chemicon), 1:500; *NSE* (neuron-specific enolase), rb pc ab (# AB951; Chemicon), 1:2000; *S100ß*, rb pc ab (# ab 7853–500; Abcam), 1:2,000; *S100ß*, rb pc ab (# 15146-1-AP; Proteintech), 1:1000; *VGAT* (vesicular GABA transporter), rb pc ab (# 131003; Synaptic Systems), 1:1000; *VGLUT1* (vesicular glutamate transporter 1), rb pc ab (# 135302; Synaptic Systems), 1:500; *VIM*, vimentin, rb pc ab (#172002; Synaptic Systems), 1:500. Fluorescent secondary antibodies were the following: Alexa 488-coupled donkey anti rb IgG, Alexa 488-coupled donkey anti ms IgG, Alexa 594-coupled donkey anti ms, and Alexa 594 donkey anti goat IgG (Molecular Probes, Invitrogen, Leiden, Netherlands), working dilution 1:500 in all cases. In double-labeling experiments, antibodies were applied sequentially and blocking steps were performed using normal serum of the host species from which the respective secondary antibody was derived. Sections were processed as described before (Araya-Callís et al. 2012). After incubation with the antibodies, sections were washed in PBS and floated/mounted on Histobond slides in distilled water, allowed to dry overnight at 4 °C and cover-slipped with mounting medium (Aqua-Polymount; Polysciences, Warrington, PA, USA).

Confocal microscopy was performed with a laser scanning microscope (LSM 5 Pascal; Carl Zeiss Microscopy, Germany) with argon (488 nm) and helium/neon (543 nm) lasers. Confocal analysis was performed in multiple-tracking mode using an Apochromat 63 oil objective (NA = 1.4) with oil immersion (Immersol, Zeiss, refractive index = 1.518). The 543-nm laser was always used with a smaller detection pinhole diameter than the 488-nm laser to produce optical sections of comparable thickness for co-localization analysis.

## Results

### Overall pattern of NDRG2 immunoreactivity

**Fig. 1:**
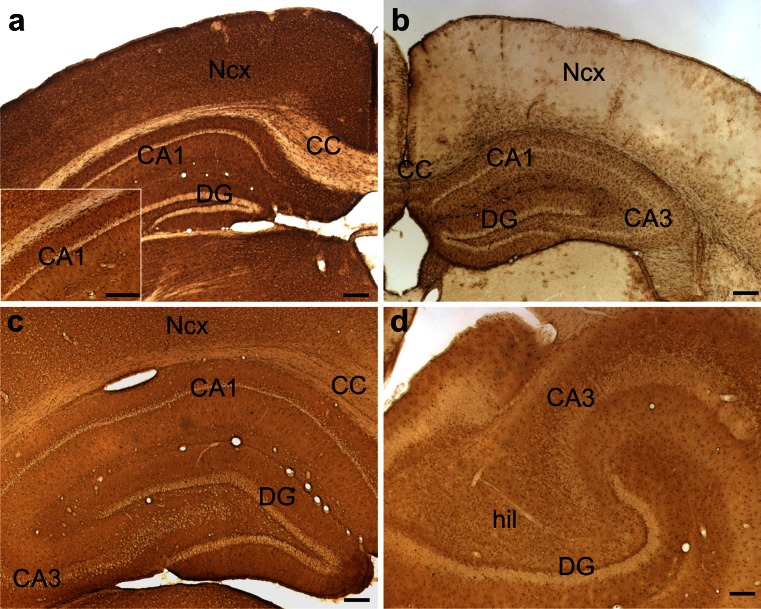
Overview of NDRG2 and GFAP immunoreactivity in brains of mouse, rat and marmoset. Sections from the level of the hippocampus were immunohistochemically processed for light microscopy. **a** Mouse, NDRG2 immunoreactivity; the *inset* shows hippocampal area CA1 at a higher magnification. **b** Mouse, GFAP immunoreactivity; note that NDRG2 immunoreactivity (**a**) is more evenly distributed throughout gray matter areas than GFAP immunoreactivity (**b**). **c** Rat, NDRG2 immunoreactivity. **d** Marmoset, NDRG2 immunoreactivity. *CA1*, *CA3* cornu ammonis regions 1 and 3; *CC* corpus callosum; *DG* dentate gyrus; *hil* hilus; *Ncx* neocortex. *Calibration bars* 200 μm

Brain sections that were immunohistochemically processed for light microscopy show that NDRG2 immunoreactive cells are more evenly distributed than GFAP-positive cells (Fig. 1, Table 1). A section from the mouse brain reveals a quite homogeneous NDRG2 staining pattern generated by the immunoreactive cell bodies. The staining patterns of the hippocampal formation are similar in mouse and rat (Fig. 1), as well as in the tree shrew (not shown). Light microscopy shows a light brown background, which is due to a large number of fine immunoreactive processes that can be distinguished when labeled for immunofluorescence in the confocal microscope (see below). White matter regions such as the corpus callosum display low levels of NDRG2 immunoreactivity, while layers with accumulated neurons (e.g., pyramidal neuron layer in CA1, granule cell layer of the dentate gyrus) are unstained. In the rat brain, the staining appears even more homogenous than in the mouse and a similar NDRG2 staining pattern is found in marmoset brain sections (Fig. 1; Supplementary Figure S1). The human brain stem shows a similar labeling as the mouse brain stem with a dense array of NDRG2 immunoreactive fibers and some cell bodies (Supplementary Figure S1). Both NDRG2 antibodies used in the present study generated the same staining pattern and there was total co-localization in double-immunofluorescence experiments (not shown).

**Table 1:** Distribution of NDRG2 in comparison with other cell markers

Antigen 1	Antigen 2	Brain regions analyzed	Species analyzed	Results
NDRG2	–	All regions	Mouse, rat, tree shrew, marmoset, human	Astrocytes are NDRG2+; distribution of NDRG2+ cells more homogeneous than distribution of GFAP+ cells
NDRG2	GFAP	All regions	Mouse, rat, tree shrew, marmoset	Co-localization in most astrocytes; NDRG2 in cytosol of cell bodies and astrocytic processes, GFAP in intermediate filaments
NDRG2	GFAP	Caudate nucleus	Rat, tree shrew, marmoset	Moderate density of NDRG2+ cells, low density of GFAP+ cells
NDRG2	GFAP	White matter	Rat, tree shrew, marmoset	Low density of NDRG2+ cells, moderate density of GFAP+ cells
NDRG2	S100ß	Gray matter areas	Rat	Co-localization in astrocytes
NDRG2	S100ß	White matter	Rat	Co-localization in a subpopulation of astrocytes
NDRG2	AQP4	All regions	Rat, tree shrew	Partial co-localization
NDRG2	NG2	All regions	Rat, tree shrew	No co-localization
NDRG2	Iba1	All regions	Rat, tree shrew	No co-localization
NDRG2	Nestin	Lesion in the neocortex; dentate gyrus	Marmoset	No co-localization
NDRG2	VIM	Lesion in the neocortex, glial scar	Marmoset	No co-localization
NDRG2	–	Retina	Tree shrew, marmoset	No NDRG2 in Müller glia; low NDRG2 expression in ganglion cell layer
NDRG2	NSE^a^	All regions	Rat, tree shrew, marmoset	No co-localization
NDRG2	MAP2^a^	All regions	Rat, tree shrew	No co-localization
NDRG2	VGlut1^a^	Cerebellum, hippocampus	Rat	No co-localization; Bergmann glia cells are NDRG2+; association of NDRG2+ processes with VGlut1+ terminals
NDRG2	VGAT	Cerebellum, hippocampus	Rat	No co-localization; association of NDRG2+ astrocytic processes with VGAT+ terminals

*AQP4* aquaporin 4; *GFAP* glial fibrillary acidic protein; *Iba1* ionized calcium-binding adapter molecule 1; *NG2* chondroitin sulfate proteoglycan 4; *NSE* neuron specific enolase; *MAP2* microtubule-associated protein 2; *VGAT* vesicular *GABA* transporter; *VGlut1* vesicular glutamate transporter 1; *Vim* vimentin; + immunopositive

^a^Neuronal marker

### NDRG2 compared to GFAP immunofluorescence

**Fig. 2:**
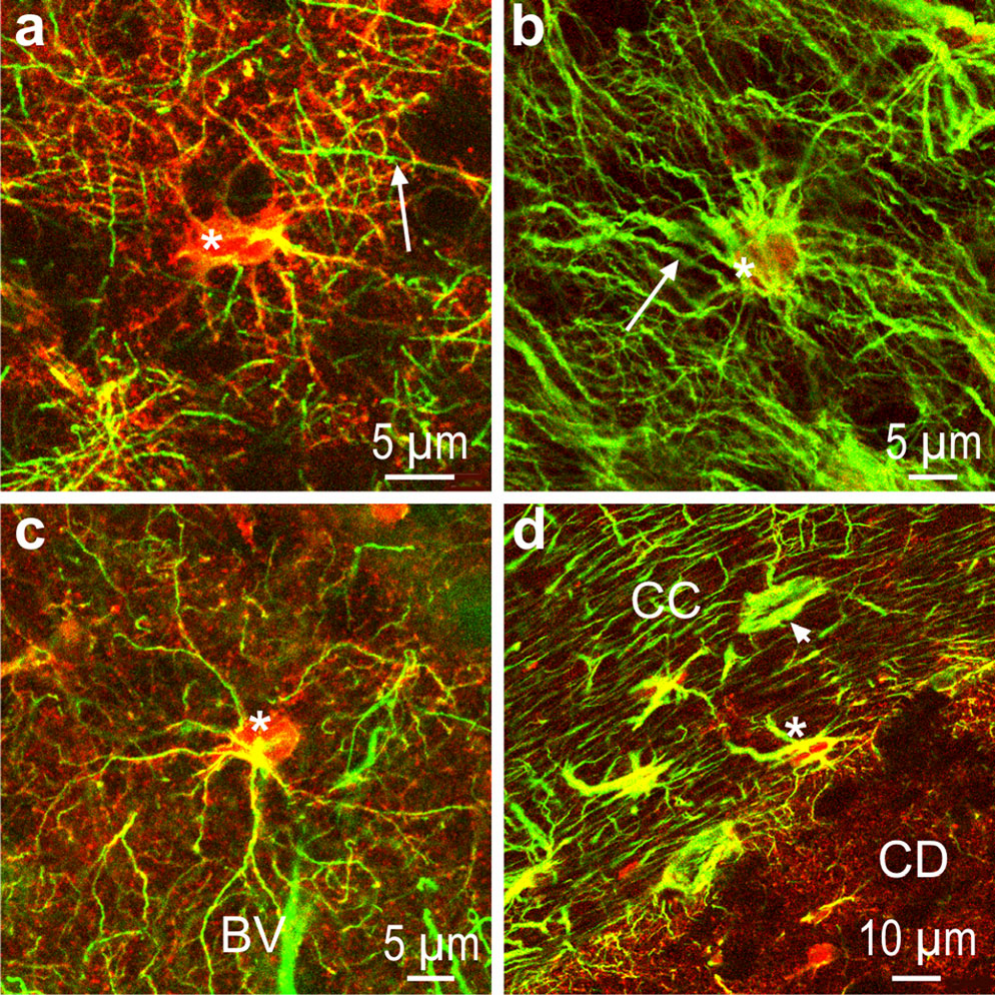
NDRG2 (*red*) and GFAP (*green*) immunoreactivity in marmoset (**a**, **b**, **d**) and tree shrew (**c**). **a** Caudate nucleus; note the strong NDRG2 immunoreactivity of the astrocytic cell body (*asterisk*) and the array of fine fibers in the surroundings of the cell; *arrow* denotes a GFAP immunoreactive fiber. **b** Cortical layer I; only a few cell bodies (*asterisk*) display NDRG2 immunoreactivity whereas GFAP-positive fibers (*arrow*) are abundant. **c** Cortical layer III; an immunoreactive NDRG2-positive astrocyte (*asterisk*) is located close to a blood vessel (*BV*). **d** Border between corpus callosum (*CC*) and caudate nucleus (*CD*); note that in the CC, there are cells stained for both antigens (*asterisk*) as well as cells in which GFAP dominates (*arrow head*). Very low GFAP immunoreactivity is found in the CD

Astrocytes are differentially labeled by antibodies against NDRG2 and GFAP, respectively (Fig. 2, Table 1). There are cell bodies in the caudate nucleus that show intense NDRG2 but little GFAP (Fig. 2a). In cortical layer III, there are cells whose entire cell bodies and processes contain NDRG2 but only a few GFAP-positive intermediate filaments (Fig. 2c). In contrast, the cortical layer I is characterized by a dense pattern of GFAP-positive fibers with little NDRG2 confined to the cell bodies (Fig. 2b). The fibrous astrocytes in the corpus callosum contain either both antigens or predominantly GFAP (Fig. 2d). The presence of cells showing no or only partial co-localization indicates that the two proteins are either expressed in different cell populations or in astrocytes of distinct physiological/metabolic states.

### Glial scar

**Fig. 3:**
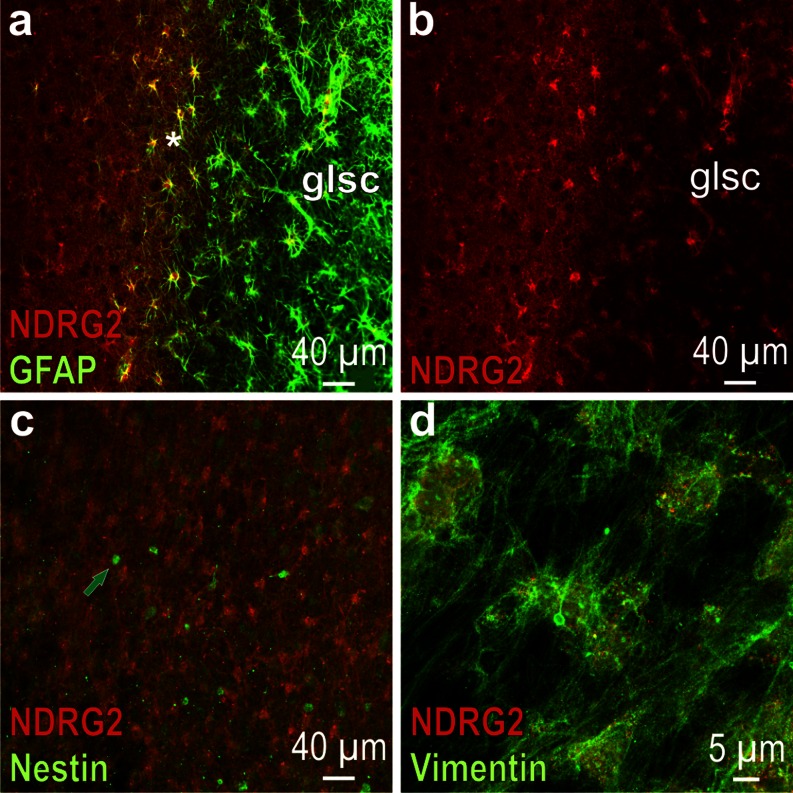
Glial scar at a trauma-induced lesion in the marmoset neocortex. **a** Merged NDRG2 and GFAP immunofluorescence; GFAP-positive cells are accumulated in the area of the glial scar (*glsc*); note the narrow zone where cells are immunoreactive for both NDRG2 and GFAP (*asterisk*). **b** Red NDRG2 immunofluorescence (channel corresponding to **a**); within the glial scar, there are only a few cells that express NDRG2 (*right*). **c** Merged NDRG2 and nestin immunofluorescence; the nestin immunoreactive cells (*arrow*) in the area of the glial scar do not express NDRG2. **d** (merged): Vimentin and NDRG2 at the surface of the glial scar; note that there is a fine vimentin immunoreactive fiber network and almost no NDRG2

To test whether NDRG2 is expressed in reactive astrocytes, sections from a marmoset with a trauma-induced lesion in the neocortex were analyzed. In the area around the lesion, three zones can be depicted: (1) the zone directly at the lesion, which represents the glial scar and is rich in GFAP but contains almost no NDRG2 (Fig. 3, right side in a, b); (2) the zone in the periphery of the scar where there is very little GFAP but pronounced NDRG2 expression (Fig. 3, left side in a, b) and (3) a narrow band between the scar and the periphery in which cells show immunoreactivity for both antigens (Fig. 3a, middle part). It thus appears that astrocytes forming the glial scar contain very little NDRG2, which indicates that expression of this protein is reduced in reactive astrocytes. Moreover, there is almost no NDRG2 in the cells at the surface of the lesion where vimentin immunoreactivity is strong (Fig. 3d). Some cells in the zone of the glial scar express nestin but also these cells show no NDRG2 (Fig. 3c).

### NDRG2 and other glial or neuronal markers

**Fig. 4:**
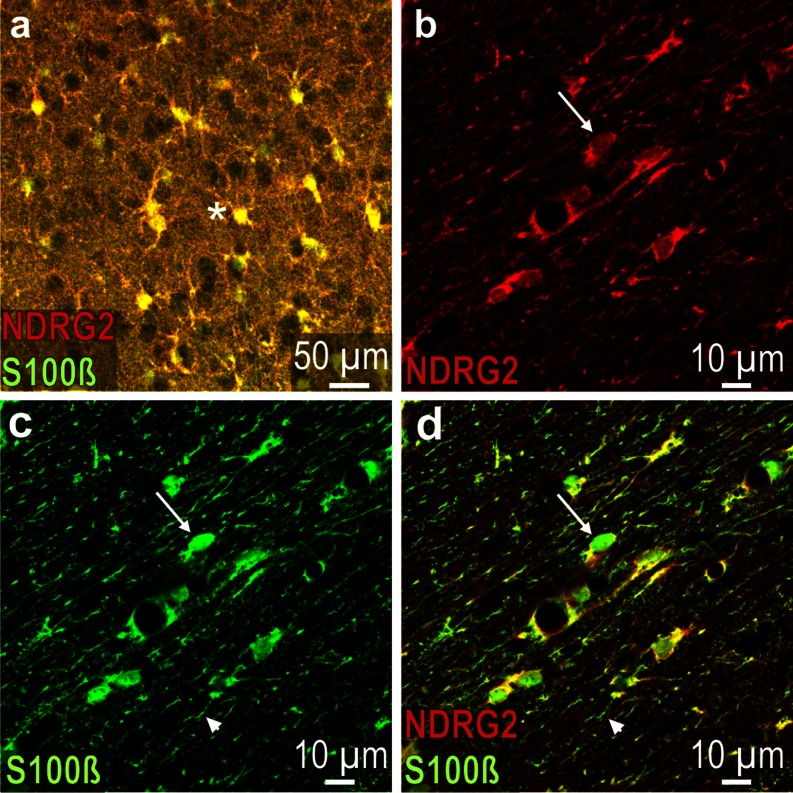
S100ß and NDRG2 immunoreactivity in the rat brain. **a** (merged) Neocortex layer III; most labeled cells (*asterisk*) are immunoreactive for both antigens (*yellow*). **b** (red channel) Corpus callosum; NDRG2 expression in some cells is relatively weak (*arrow*). **c** Green channel (corresponding to **b**): The cell that is also indicated in (**b**) (*arrow*) shows a pronounced S100ß expression; *arrowhead* denotes S100ß immunoreactive fiber. **d** (merged) The cell that is also indicated in (**b** and **c**) shows strong S100ß but weak NDRG2 immunoreactivity; *arrowhead* denotes S100ß immunoreactive fiber that does not contain NDRG2

NDRG2 co-localizes with S100ß in most gray matter astrocytes as shown in the neocortex (Fig. 4). In contrast, in white matter areas, there is only partial co-localization in distinct cells. The subcellular localization of the two antigens differs as, e.g., demonstrated in the corpus callosum where certain cell bodies are entirely stained for S100ß but only partially for NDRG2 and some astrocytic processes display solely S100ß immunoreactivity (Fig. 4b–d). S100ß is also strongly expressed in the cells forming the glial scar, which display no NDRG2 immunoreactivity (Supplementary Fig. S3d).

**Fig. 5:**
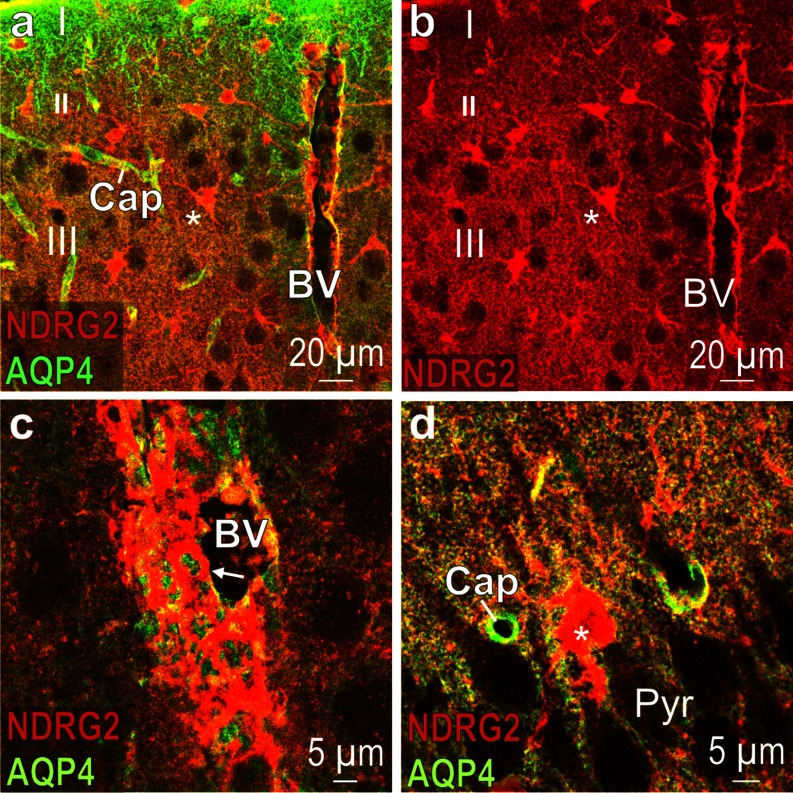
Partial co-localization of NDRG2 and AQP4 immunoreactivity. **a** (*merged*) Tree shrew neocortical layers I-III; note the array of punctual AQP4 immunoreactivity and some NDRG2-positive cell bodies (*asterisk*); the wall of a large blood vessel (*BV*) displays NDRG2 immunoreactive structures; *Cap* capillary. **b** Red channel corresponding to (**a**). **c** (merged) High magnification of a blood vessel in tree shrew cortical layer III; *arrow* indicates astrocytic process at the wall of the blood vessel; note that there is only partial co-localization with AQP4 (*yellow* structures). **d** (merged) Rat hippocampal region CA3; note the NDRG2 immunoreactive cell body (*asterisk*); co-localization with AQP4 is indicated by the *fine yellow line* that surrounds the NDRG2 immunoreactive cell body. *Pyr* pyramidal neuron layer

The water channel aquaporin 4 (AQP4), which is located in the astrocytic plasma membrane and is concentrated in endfeet, co-localizes partially with the cytosolic protein NDRG2 (Fig. 5). Small capillaries are easily distinguishable because of their AQP4-positive astrocytic endfeet, which terminate on the walls of these capillaries (Fig. 5a, d). The wall of a large blood vessel shows a dense pattern of NDRG2 immunoreactive astrocytic processes (Fig. 5c). A fine line of yellow fluorescence around an astrocyte in hippocampal region CA3 indicates a close vicinity of the NDRG2 and the S100ß antigen.

NDRG2 does not co-localize with the neuronal markers NSE (neuron-specific enolase) and MAP2 (microtubule-associated protein 2) (Supplementary Figure S2). There is also no co-localization with the ‘neuroglia’ marker NG2 or with the microglia marker Iba1 (ionized calcium-binding adapter molecule 1) (Supplementary Figure S3a, c). In brains of adult marmosets, nestin immunoreactive fibers were also detected close to the brain surface at the ventromedial pole of the dentate gyrus. However, no co-localization with NDRG2 was observed indicating that the nestin-positive fibers do not belong to NDRG2 cells (Supplementary Figure S3b).

### Astrocytic processes at nerve terminals

**Fig. 6:**
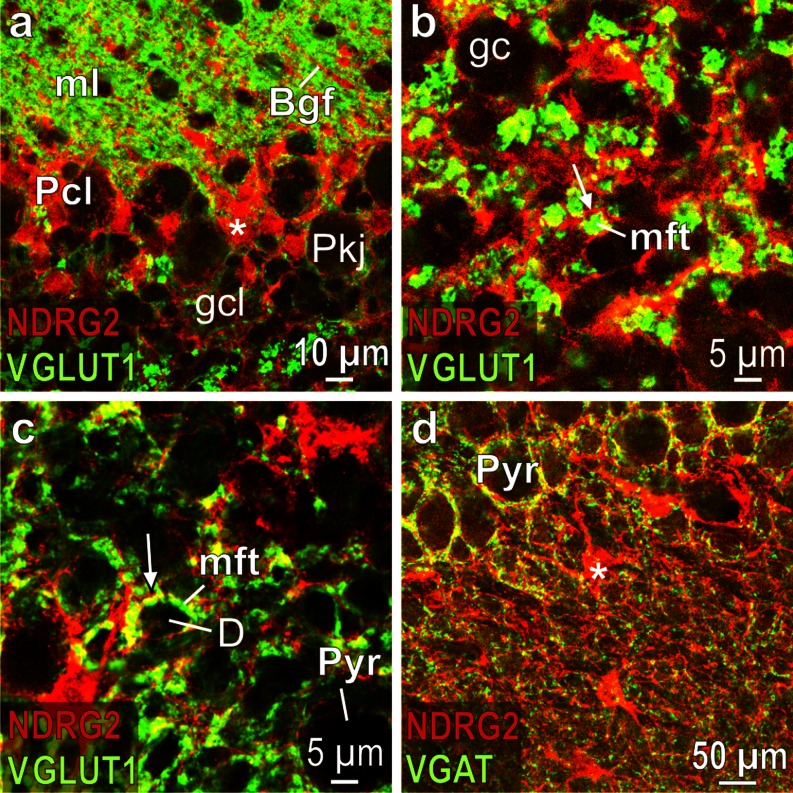
Glia cells in rat cerebellum and hippocampus. **a** (merged) Cerebellar Purkinje cell layer (*Pcl*); note the strongly NDRG2 immunoreactive Bergmann glia cell bodies (*asterisk*) that are located between the unlabeled Purkinje neurons (*Pkj*); Bergmann glial fibers (*Bgf*; *red*) in the molecular layer (*ml*) are associated with the glutamatergic parallel fibers (*green*); *gcl* granule cell layer. **b** (merged) Layer of granule cells (*gc*) in the cerebellum; note the NDRG2 process (*arrow*), which is associated with a VGLUT1 immunoreactive mossy fiber terminal (*mft*). **c** (merged) Hippocampal stratum lucidum; note the fine NDRG2 positive process (*arrow*), which is associated with the giant VGLUT1 immunoreactive mossy fiber terminal (*mft*) that synapses on dendrites (*D*) of pyramidal neurons (*Pyr*). **d** (merged) Hippocampal stratum lucidum; note that fine NDRG2 positive processes are associated with VGAT immunoreactive nerve terminals; *asterisk* indicates NDRG2 immunoreactive cell body

Bergmann glial cells strongly express NDRG2 (Fig. 6). In the cerebellar granule cell layer, the immunoreactive processes of astrocytes are located close to the mossy fiber terminals that are stained by the antibody against the vesicular glutamate transporter 1 (VGLUT1) (Fig. 6b). Similarly, NDRG2 containing astrocytic processes are associated with glutamatergic nerve terminals in the hippocampal stratum lucidum (Fig. 6c). NDRG2-positive processes are also associated with GABAergic nerve terminals, which are stained for the vesicular GABA transporter (VGAT) as shown in the stratum lucidum (Fig. 6d, Table 1).

### Retina

**Fig. 7:**
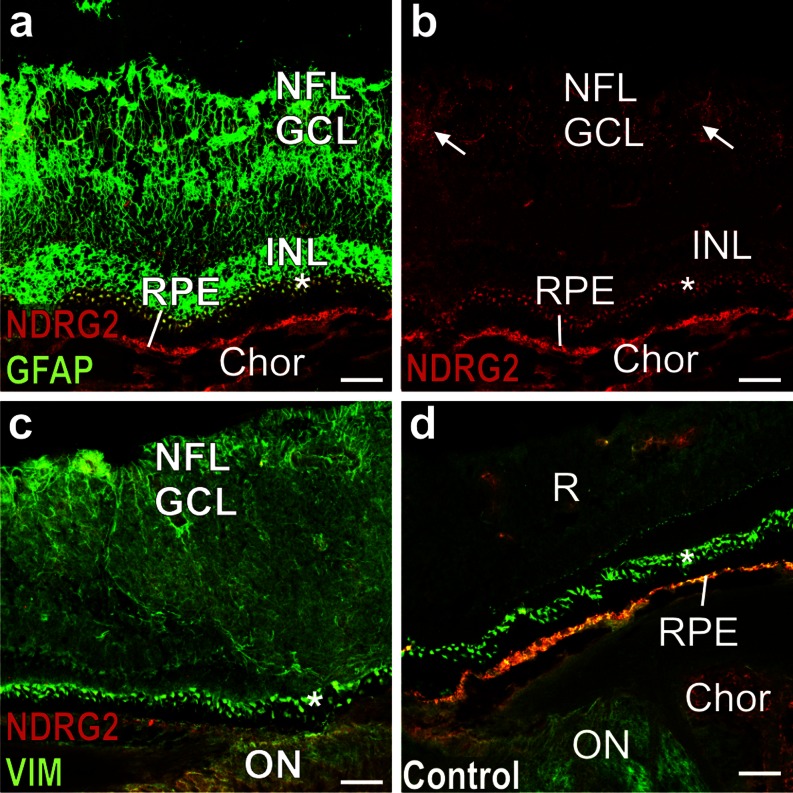
NDRG2 and GFAP in the marmoset retina. **a** (merged) NDRG2 and GFAP immunoreactivity. **b** (red channel corresponding to **a**) *Arrows* denote faint NDRG2 immunoreactivity. **c** (merged) NDRG2 and vimentin (*VIM*) immunoreactivity. **d** (merged) Control section incubated with just the secondary antibodies. *Asterisks* denote an outer nuclear layer that displays nonspecific immunofluorescence. *Chor* choroid; *GCL* ganglion cell layer; *INL* internal nuclear layer; *NFL* nerve fiber layer; *ON* optic nerve; *R* retina (all layers); *RPE* retinal pigment epithelium. *Calibration bars* 50 μm

Preliminary experiments revealed that GFAP and NDRG2 immunoreactivity in the retina are fixation-sensitive. For this reason, we generated sections from freshly frozen marmoset retinae and post-fixed them only briefly with 2 % PFA (see “Materials and methods”). With this approach, GFAP and vimentin immunoreactivity can be detected in the retinal layers (Fig. 7). Clusters of GFAP positive fibers are present in the inner nuclear layer (INL), which contains the Müller cells bodies as well as in the nerve fiber (NFL) and ganglion cell layer (GCL) (Fig. 7a). In contrast, no NDRG2 immunoreactivity is found in the INL. Furthermore, only traces of NDRG2 immunofluorescence are visible in the NFL/GCL, possibly representing small amounts of the antigen in astrocytes (Fig. 7b). Only connective tissue in the choroid displays pronounced NDRG2 immunoreactivity. Vimentin is present throughout all layers of the retina (Fig. 7c). However, the strong fluorescence of the retinal pigment epithelium (RPE) and of the adjacent outer nuclear layer (asterisk) is nonspecific as it is also visible in control sections incubated with only the secondary antibodies (Fig. 7d).

## Discussion

The present data reveal NDRG2 expression in a large population of brain astrocytes. These cells are relatively homogeneously distributed throughout gray matter areas. NDRG2 expressing astrocytes also contain S100ß but the NDRG2 cells appear to represent a different population from the S100ß cells. Fibrous astrocytes of white matter express NDRG2 less strongly than GFAP. No or very low NDRG2 is found in reactive astrocytes. Our findings thus show that NDRG2 is a suitable marker for mature, non-reactive brain astrocytes.

After injury to the brain, mature astrocytes in the area of the lesion can de-differentiate to cells that form a scar (Wachter et al. 2010). The present data reveal that NDRG2 is almost absent from the astrocytes that form the glial scar around a trauma-induced lesion in the marmoset neocortex, indicating that NDRG2 expression is downregulated in reactive astrocytes. It has been proposed that, in the area of a lesion, inflammatory mediators derived from blood-borne and injury-associated cells regulate astroglial transcription, proliferation and function (Wanner et al. 2013). Serious insults such as trauma, ischemia, or autoimmune inflammation induce proliferation of astrocytes and other cells, which leads to formation of the glial scar (Sofroniew 2009). Expression of the progenitor and radial glial marker vimentin and of GFAP strongly increases after injury to the brain (Bramanti et al. 2010; Kim et al. 2012). The population of astrocytes in the area of a glial scar is heterogeneous and varies with the distance from the scar. At least two categories of reactive astrocytes have been observed in such an area: (1) newly proliferated, elongated astrocytes that form the scar and (2) hypertrophic stellate, reactive astrocytes that do not proliferate and derive from local astroglia (Wanner et al. 2013). Accordingly, in the marmoset neocortex, cells expressing vimentin were detected directly at the lesion. Also, cell bodies immunoreactive for nestin, the stem cell-associated intermediate filament protein that is expressed during development, have been observed at a glial scar (Pekny and Pekna 2004). In the present study, nestin-positive cells were located at some distance from the lesion. None of these cells expressed NDRG2 and, furthermore, NDRG2 was not found in the strongly GFAP immunoreactive astrocytes. Co-localization of NDRG2 and GFAP was only detected in cells within a narrow zone between the area of the scar and the surrounding unaffected tissue. It appears that the astrocytes in that narrow zone are in a transient situation between a quiescent and reactive state. They possibly represent resident astrocytes that are about to de-differentiate in the process of astrogliosis (Wachter et al. 2010).

As a structural protein of intermediate filaments (IF), GFAP may play a role in IF rearrangements during astrocyte migration (Lepekhin et al. 2001). Over-expression of the protein in mouse models of Alexander Disease induced hypertrophic astrocytes that had lost their bushy appearance due to a lack of fine distal processes (Messing and Brenner 2003; Sosunov et al. 2013). GFAP in reactive astrocytes is thus associated with proliferation and allows cell migration in the course of brain tissue regeneration. In contrast, NDRG may suppress proliferation and may stabilize cell morphology (Takeichi et al. 2011). It is also interesting to note that, in vitro*, Ndrg2* gene induction by a growth factor enhanced sprouting and extension of cell processes in another cell type, PC 12 cells (Takahashi et al. 2005). Furthermore, studies on skeletal muscle tissue provided indications that increased levels of NDRG2 do not stimulate proliferation but are related to homeostasis of the cells, in this case myotube function (Foletta et al. 2009). One may speculate that brain astrocytes with normal levels of NDRG2 are in a ‘non-reactive’ state that allows their homeostatic functioning within the neural network. However, it remains to be determined whether this ‘non-reactive’ state is related to the mechanisms through which NDRG2 suppresses cell proliferation.

Expression of the calcium binding protein S100ß characterizes cells that lose their neural stem cell potential and acquire a mature developmental stage (Raponi et al. 2007). The present data confirm previous findings showing that S100ß is expressed in a large population of astrocytes (Adami et al. 2001). In the frontal brain, NDRG2 co-localizes with S100ß in the majority of cells. However, the subcellular localization of the two proteins differs in that NDRG2 is only found in the cytosol whereas S100ß is also associated with subcellular membranes and the cytoskeleton (Adami et al. 2001). Another difference between the two proteins is that S100ß is increased in reactive astrocytes whereas NDRG2 is not. However, S100ß antibodies may not only label astrocytes but also certain neurons, presumably because of cross-reactivity of respective antibodies with different members of the S100 gene family (Rickmann and Wolff 1995a). Coinciding with this, we observed strong S100ß immunoreactivity in certain brain stem neurons such as the motor neurons of the trigeminal nucleus (not shown). A further complication in experiments with S100ß antibodies is that the intensity of the immunoreactvity is sensitive to the tissue fixation (Rickmann and Wolff 1995b). In view of such potential problems with S100ß antibodies, one may regard NDRG2 as a more reliable marker for mature brain astrocytes.

The present study shows that NDRG2 is strongly expressed in Bergmann glia cells that are known to interact with the Purkinje neurons (Bellamy 2006). We visualized glutamatergic and GABAergic nerve terminals with antibodies against the vesicular glutamate transporter and the vesicular GABA transporter, respectively. In the confocal microscope, NDRG2 was found to be closely associated with these nerve terminal markers indicating that the protein is present in the fine distal astrocytic processes that contact nerve terminal structures including synapses (Araque et al. 1999; Haydon 2001). In contrast, GFAP that constitutes only ∼15 % of the total volume of an astrocyte is not found in the distal astrocytic processes that contact synapses (Bushong et al. 2002; Lavialle et al. 2011).

It is interesting to note that NDRG2 has been shown to interact with a subunit of sodium-potassium ATPase, at least in epithelial cells (Li et al. 2011). The astrocytic ATPase is important for the regulation of neurotransmission as it restores the ion gradient necessary to drive glutamate transport (Haydon and Carmignoto 2006; Pellerin and Magistretti 1997). However, the numerous interactions between neurons and glia cells are not confined to synaptic structures but ‘convoluted cytoplasmic tongues’ of astrocytes appear to ‘branch randomly into the neuropil’ (Reichenbach et al. 2010). The cytosolic protein NDRG2 may thus be a suitable marker to visualize sites of interactions between astrocytic and neuronal structures.

The architecture of the marmoset retina has been described before (Hendrickson et al. 2006). We observed strong GFAP and vimentin staining of all retinal layers including the inner nuclear layer that contains the somata of Müller cells (Bringmann et al. 2013). However, the NDRG2 antibody did not label the Müller cells. Only the nerve fiber/ganglion cell layer showed a minor immunofluorescence, probably related to astrocytes. It is interesting to note that NDRG2, which is a suppressor of cell proliferation, displays such a low level of expression in the retina, a tissue with a high potential of cell regeneration (Lewis and Fisher 2003). This finding provides further evidence that NDRG2 is not expressed in proliferating glia cells.

In conclusion, our results show that NDRG2 is a marker for mature, nonreactive, non-proliferating astrocytes. Antibodies against this marker protein may be suitable for a morphological analysis of the respective subpopulation of astrocytes.

## Electronic supplementary material

Below is the link to the electronic supplementary material.

Supplementary Figure S1 (PDF 149 kb)

Supplementary Figure S2 (PDF 46 kb)

Supplementary Figure S3 (PDF 283 kb)
