# Supplementary material for: NDRG2 as a marker protein for brain astrocytes
Source: Cell Tissue Res. 2014 May 10;357(1):31–41. doi: 10.1007/s00441-014-1837-5 (PMC4077251; doi:10.1007/s00441-014-1837-5)
Supplement: Supplementary file 1 — (PDF 149 kb) [file 441_2014_1837_MOESM1_ESM.pdf]

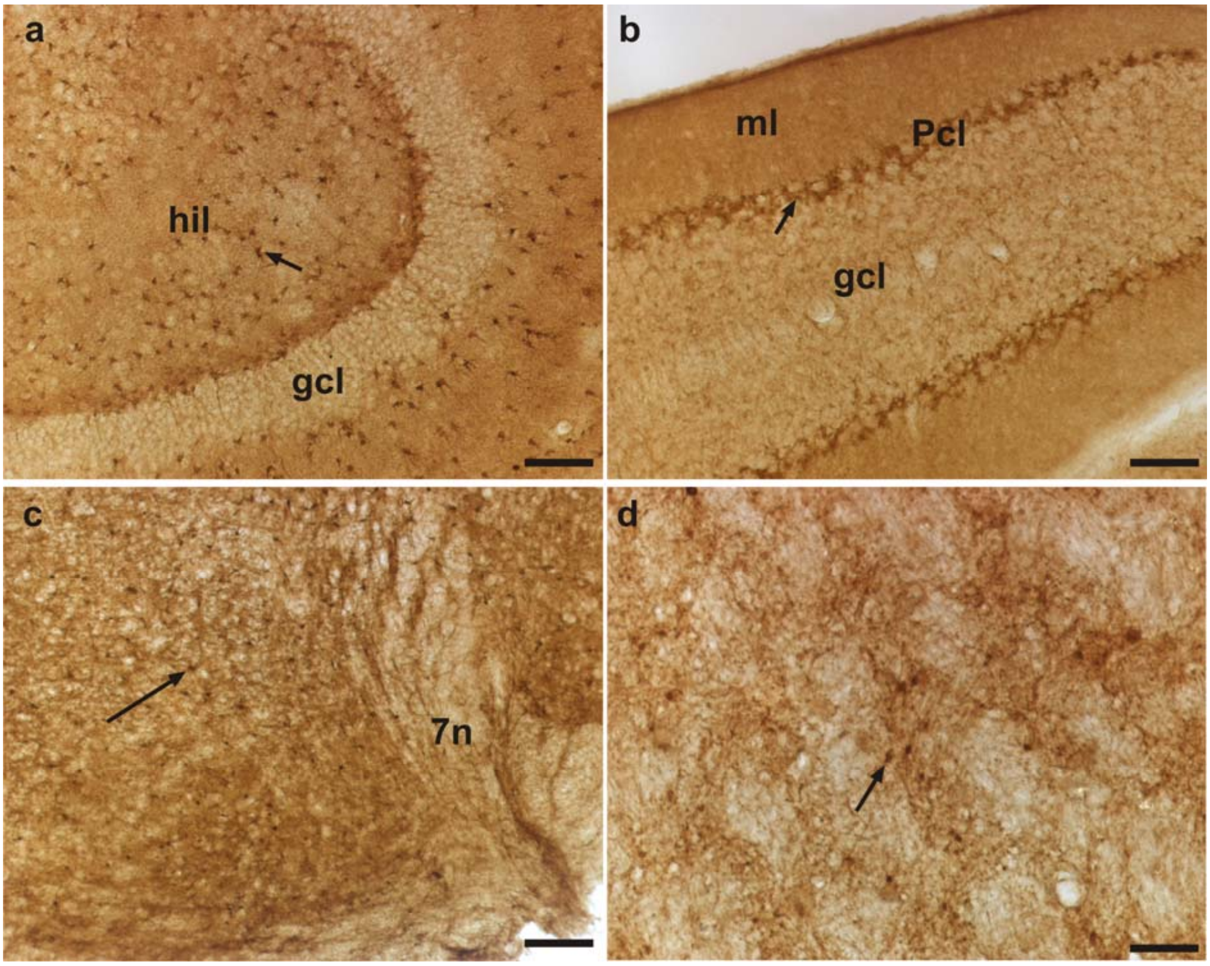

Figure S1

NDRG2 in brain sections from different species. Sections were incubated with goat anti NDRG2 antibody and immunohistochemically processed for light microscopy as described in Materials and methods. In **a:** Marmoset dentate gyrus. In **b:** Tree shrew cerebellum: Note the strongly stained astrocytes in the Purkinje cell layer (Pcl). In **c**: Mouse brain stem with facial nerve (7n). In **d**: Human formatio reticularis. Abbreviations: gcl, granule cell layer; hil, hilus; ml, molecular layer. Arrows denote NDRG2 immunopositive astrocytes. Calibration bars represent 100 µm in **a-c**, 25 µm in **d**
