# Supplementary material for: NDRG2 as a marker protein for brain astrocytes
Source: Cell Tissue Res. 2014 May 10;357(1):31–41. doi: 10.1007/s00441-014-1837-5 (PMC4077251; doi:10.1007/s00441-014-1837-5)
Supplement: Supplementary file 2 — (PDF 46 kb) [file 441_2014_1837_MOESM2_ESM.pdf]

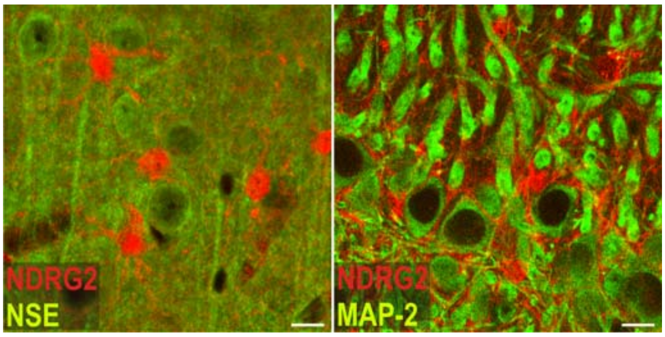

Figure S2

NDRG2 does not co-localize with the neuronal markers NSE (neuron specific enolase) and MAP2 (microtubule-associated protein 2). Left, rat neocortex; right, rat CA3. Double immunofluorescence experiments were performed with antibodies goat anti NDRG2, rabbit anti NSE and/or rabbit anti MAP2 as described in Materials and methods. Calibration bars: 10 µm
