# Supplementary material for: NDRG2 as a marker protein for brain astrocytes
Source: Cell Tissue Res. 2014 May 10;357(1):31–41. doi: 10.1007/s00441-014-1837-5 (PMC4077251; doi:10.1007/s00441-014-1837-5)
Supplement: Supplementary file 3 — (PDF 283 kb) [file 441_2014_1837_MOESM3_ESM.pdf]

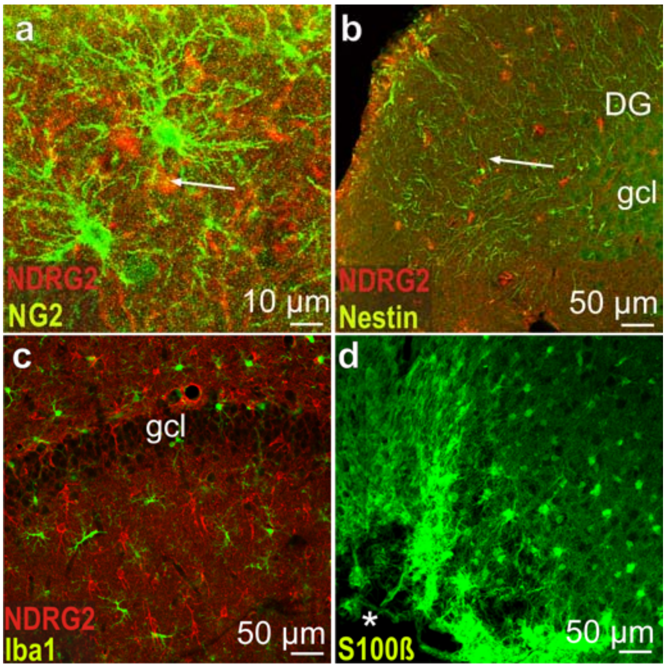

Figure S3

NDRG2 and other glial markers. Sections were incubated with antibodies and processed to visualize immunofluorescence as described in Materials and methods. In **a**: NG2 and NDRG2 immunoreactivity in rat cortical layer I-II. Note that there is no co-localization of the two marker proteins in the cytoplasma of the cells. However, the tiny yellow dots (arrow) indicate that astrocytes and NG2 cells contact each other, presumably via their fine distal processes. In **b**: Nestin and NDRG2 in the ventromedial pole of the marmoset dentate gyrus (DG). There is no co-localization of the nestin immunopositive fibers (arrow) and the NDRG2 cells; gcl, granule cell layer. In **c**: Iba1 and NDRG2 in rat dentate gyrus. There is no co-localization of the microglia marker Iba1 and NDRG2. In **d**: S100ß immunoreactivity at a lesion (asterisk) in marmoset neocortex. Note that cells located close to the lesion strongly express S100ß
